# Cerebrospinal Fluid Extracellular Vesicles with Distinct Properties in Autoimmune Encephalitis and Herpes Simplex Encephalitis

**DOI:** 10.1007/s12035-021-02705-2

**Published:** 2022-01-27

**Authors:** Yongang Li, Jiachen Gu, Youbing Mao, Xijia Wang, Zongshan Li, Xiaomin Xu, Huimin Chen, Yaxing Gui

**Affiliations:** 1 Department of Neurology, The First People’s Hospital of Wenling, Wenling, China; 2 Department of Neurology, Shanghai General Hospital, Shanghai Jiao Tong University School of Medicine, No.86 Wujin Road, Shanghai, 200080 China; 3 Department of Neuroelectrophysiology, The First People’s Hospital of Wenling, Wenling, China; 4 Department of Neurology, School of Medicine, Sir Run Run Shaw Hospital, Zhejiang University, Hangzhou, China

**Keywords:** Exosomes, microRNAs, Autoimmune encephalitis (AE), Herpes simplex encephalitis (HSE)

## Abstract

Encephalitis mediated by autoantibodies against neuronal antigens and herpes simplex encephalitis (HSE) are seemingly separate causes of encephalopathy in adults. Autoimmune encephalitis (AE) is autoimmune in origin, and herpes simplex encephalitis is infectious. The purpose of this study was to examine the role of cerebrospinal fluid (CSF) exosomes from patients with antibody-positive AE and HSE. Towards this, exosomes were isolated from CSF from 13 patients with anti-*N*-methyl-d-aspartate receptor (NMDAR) encephalitis, 11 patients with anti-gamma-aminobutyric acid-B (GABAB) receptor encephalitis, 9 patients with anti-leucine-rich glioma-inactivated 1 (LGI1) encephalitis, and 8 patients with anti-contactin-associated protein-like 2 (CASPR2) encephalitis, and 12 control individuals negative of antibodies against neuronal autoantigens. There were ten miRNAs highly expressed in patients with anti-NMDAR encephalitis compared to those in control subjects. Eight miRNAs were found to be lower expressed in anti-NMDAR encephalitis CSF-derived exosomes. Furthermore, Kyoto Encyclopedia of Genes and Genomes (KEGG) pathways enriched by AE differential expressed exosomic miRNAs demonstrated that AE-related exosomic miRNAs may participate as a feedback regulation in cancer development. In addition, the exosome concentration in CSF of 9 HSE patients was significantly higher compared to those from 9 HSV( −) patients. This observation was consistent with the results that exosome concentration was found to be higher in the animal model which was inoculated intranasally with HSV-1 compared to controls. Furthermore, western blot demonstrated that the subunits of NMDAR, GABA_B_R, and AMPAR were detected highly expressed in exosomes derived from sera of HSV-1-treated animal model compared to controls. More importantly, exosomes isolated from CSF of HSE patients contained higher expression levels of two miRNAs encoded by HSV, miR-H2-3p, and miR-H4-3p compared to those from HSV( −) patients. In summary, HSV may trigger brain autoimmunity in HSE by presentation of surface autoantigens via exosomes.

**Supplementary Information:**

The online version contains supplementary material available at 10.1007/s12035-021-02705-2.

## Introduction

Encephalitis is one type of brain inflammation caused by virus infection or autoimmune-mediated reactions. Several types of encephalitis, including the classic paraneoplastic encephalitis syndromes, are immune mediated and often associated with autoantibodies against autoantigens, including neuronal surface proteins [1]. Dalmau et al. reported that half of women with anti-*N*-methyl-d-aspartate receptor (NMDAR) encephalitis had underlying mature or immature ovarian teratomas [2]. Dalmau et al. also confirmed that ovarian teratomas containing nervous tissue exhibited NMDAR subunits [2]. Several other autoantibodies against multiple neuronal autoantigens have been discovered in autoimmune encephalitis (AE) disease [3–5], including anti-gamma-aminobutyric acid-B receptor (GABA_B_R) encephalitis, anti-leucine-rich glioma-inactivated 1 (LGI1) encephalitis, and anti-contactin-associated protein-like 2 (CASPR2) encephalitis. The most frequent neoplasms associated with paraneoplastic limbic encephalitis are small cell lung cancer (SCLC), testicular tumors, and Hodgkin lymphoma. The type of associated autoantibody varies with tumor type. Most SCLC patients have anti-Hu (also known as anti-neuronal nuclear antibody type 1 [ANNA-1]) or CV2/collapsin-responsive mediator protein-5 (CRMP5) antibodies in their serum and cerebrospinal fluid (CSF) [6, 7], and these patients are more likely to develop other manifestations of paraneoplastic encephalomyelitis. Anti-NMDAR encephalitis can be associated with an underlying tumor that stimulates the production of anti-NMDAR antibodies. Ovarian teratoma is the most commonly associated tumor and is reported to be present in over half of adult females [8]. Half of adult cases with anti-GABA_B_ receptor encephalitis have associated small cell lung carcinoma. Most adult cases with anti-AMPA receptor encephalitis are associated with neoplasms (including SCLC, thymoma, ovarian teratoma, and breast cancer) [9, 10].

Herpes simplex virus (HSV)-1 is neurotropic and highly destructive when it infects the brain, and it was reported to be associated with the development of encephalitis [11]. HSV encephalitis (HSE) has a critical mortality rate of nearly 30% if left untreated [12, 13]. The consequence of HSE despite treatment leads to devastating sequelae, including memory deficits and seizures [12, 13]. Although some patients were negative for the detection of HSV after treatment, retrospective analyses [14, 15] demonstrated that anti-NMDAR antibodies were detected in the sera and/or CSF, suggesting that early viral infection with HSV sowed the seeds of autoimmune encephalitis. Nearly 30% of patients with HSE develop autoimmune encephalitis, usually associated with the development of anti-NMDAR antibodies in a prospective study [16]. A study from Linnoila et al. reported an endogenous rodent model of post-HSE NMDAR encephalitis to clinically phenocopy the pathogenesis of autoimmune encephalitis [17]. The authors further found that anti-NMDAR antibodies were detected in the serum isolated from mice inoculated intranasally with HSV-1 [17]. However, the causality between HSV infection and the development of anti-NMDAR antibody encephalitis remains elusive.

Exosomes are membrane-bound nanostructure vesicles shed by endocytic pathways [18, 19]. Most cells in the CNS, including neurons, astrocytes, oligodendrocytes, and microglia, shed exosomes under both normal and pathological conditions [20] [21]. Exosomes are confirmed to mediate several biological/cellular processes required for normal brain function [22–24], as well as the pathogenesis of some neuroinflammatory disorders [25] and neurodegenerative diseases [26–28]. Exosomes are also found to serve as carriers of misfolded and pathogenic proteins [25, 29]. Aberrantly expressed cellular miRNAs were further validated to be selectively packaged and transported in exosomes to neighboring cells [21, 30]. Recent studies from our laboratory demonstrated that exosomes were present in the CSF of AE patients. In addition, specific neuronal autoantigens, including NMDAR, GABA_B_R, LGI1, CASPR2, and AMPAR, were found to be expressed in CSF- or serum-derived exosomes from AE patients. Importantly, we found that AE-derived exosome immunization in mice led to an increased frequency of neuronal autoantigen–specific IL-17 and IFN-γ [31]. Therefore, these data indicated that exosomal presentation of neuronal autoantigens would cause autoimmunity, which is usually recognized as a novel and critical pathway in AE pathogenesis.

Thus far, noncoding RNAs in CSF exosomes from AE have not been explored. Toward this, we attempted to determine the miRNA profiles in exosomes secreted from local sites in AE brain, and we correlated the dysregulation of miRNAs with clinical features such as paraneoplastic occurrence. In addition, the mechanisms that allow the establishment of HSV-mediated encephalitis remain poorly understood. We hypothesized that HSV infection increased the presentation of surface autoantigens via exosomes, which would further cause the autoimmune response during HSE development. To this end, we investigated the exosome cargo including neuronal antigens and miRNAs encoded by HSV in HSE patients as well as the animal model of HSE.

## Materials and Methods

### Ethics and Subjects

All subjects consented to participate in the study, and the work received approval from the institutional ethics committee of Sir Run Run Shaw Hospital affiliated with Zhejiang University School of Medicine and was conducted in accordance with the tenets of the Declaration of Helsinki. AE patients were diagnosed according to the autoimmune encephalitis diagnostic criteria published in The Lancet Neurology [1]. The laboratory used indirect immunofluorescence assay for CSF and/or sera antibody detection as previously reported [1]. The control individuals were diagnosed without antibodies against neuronal autoantigens. All the AE patients and control individuals show herpes simplex virus type 1 (HSV-1) PCR negative. CSF samples from these subjects were further subject to identify CSF-specific oligoclonal IgG bands (OCBs). Positive OCBs (IgG) were identified in 53.8% of patients with anti-NMDAR (7/13) and 54.5% anti-GABA_b_R (6/11); 4 patient with anti-LGI1 antibodies (44.4%) and 3 patients with anti-CASPR2 antibodies (37.5%) revealed positive OCBs. Clinical information for the discovery cohort, including demographic features and symptoms, is included in Table 1. HSE patients were diagnosed by two senior neurologists, and HSV DNA PCR from CSF samples was positive in these patients. HSV-negative controls came from the emergency department. They presented with headache and fever, and encephalitis was excluded after CSF examination and magnetic resonance imaging scanning. Clinical information of HSV-positive/negative patients is included in Table 2 and Supplemental Table 2 (HSV-negative patients).

**Table 1:** Demographic features and symptoms of cohorts with antibody-positive paraneoplastic autoimmune encephalitis

	Anti-NMDAR	Anti-GABAbR	Anti-LGI1	Anti-CASPR2	Control Subjects
No. of patients	13	11	9	8	12
Sex (F/M)	9/4	6/5	3/6	4/4	6/6
Age (year) (mean (Q1–Q3))	28 (18–51)	57 (42–72)	62 (60–72)	61 (55–69)	43 (22–63)
Neurological syndrome					
Limbic encephalitis	12	7	7	1	0
Brainstem encephalitis	1	0	1	1	0
Cerebellar degeneration	0	3	5	3	0
Peripheral nerve disorders	0	1	0	4	0
CSF analyses					
Any abnormality	10	9	7	4	0
Elevated protein	5	4	3	1	0
Lymphocytic pleocytosis	5	2	1	1	0
Oligoclonal IgG bands	7	6	4	3	0
Cancer					
Small cell lung cancer	0	2	2	0	0
Non-small cell lung cancer	0	0	2	1	0
Testicular	0	0	0	0	0
Breast	1	1	0	0	0
Gynecological	8	3	0	0	0
Hodgkin	0	0	0	0	0
No tumor	4	5	5	7	12

*Q1*, first quartile; *Q3*, third quartile

**Table 2:** Demographic features and symptoms of herpes simplex encephalitis patients

Patient no	1	2	3	4	5	6	7	8	9
Age (year)	23	18	35	29	42	27	39	26	20
Sex	Male	Male	Female	Male	Male	Female	Female	Male	Male
Neurological syndrome									
Decreased level of consciousness	+	+	-	+	-	+	+	+	-
Mental symptoms	+	-	-	+	+	+	+	-	-
Fever	+	+	+	+	+	+	+	+	+
Headache	+	+	-	+	+	+	-	+	-
Seizure	-	+	-	+	-	+	+	-	-
Focal sign	+	+	+	-	+	+	+	+	+
CSF analyses									
Increased opening pressure	-	+	-	+	+	+	+	+	-
Elevated white cells	+	+	+	+	+	+	+	+	+
Elevated red cells	-	-	+	+	-	+	-	-	-
Elevated protein	-	+	+	+	-	+	+	+	-
MRI T2 lesions									
Temporal lobe	-	+	-	-	+	+	+	-	-
Medial/inferior frontal lobe	-	-	+	+	-	-	-	+	+
Cingulate/insula cortex	+	-	+	-	-	-	-	-	+
Hippocampal	-	+	-	+	-	-	-	-	-
Other	-	-	-	-	+	-	+	-	-
HSV PCR	+	+	+	+	+	+	+	+	+

*CSF*, cerebrospinal fluid; *MRI*, magnetic resonance imaging; *HSV*, herpes simplex virus; *PCR*, polymerase chain reaction

### Isolation and Purification of Exosomes

Cerebrospinal fluid was obtained by lumbar puncture following a standard protocol. CSF exosomes were isolated from CSF samples as previously reported from our group [32]. In brief, CSF was subjected to successive centrifugations of 3,000 × *g* (15 min) and 10,000 × *g* (30 min). Exosomes were then pelleted at 100,000 × *g* for 1 h, using an SW28 rotor (Beckman, Brea, CA). Exosome pellets were further purified with sucrose cushion (30%) ultracentrifugation to eliminate protein aggregates as previously reported [33]. Exosome pellets were resuspended in 0.32 M sucrose and centrifuged at 100,000 × *g* for 1 h (SW60Ti rotor; Beckman), and then resuspended in TRIzol for mRNA extraction or denatured in the protein loading buffer.

### Transmission Electron Microscopy (TEM)

Exosome samples were first loaded onto Grids-Formvar/Carbon Coated, fixed in 2% paraformaldehyde (PFA), and washed with Gibco phosphate-buffered saline (PBS) of high purity. Samples were further processed under 2.5% glutaraldehyde fixative, washed with PBS, contrasted in 2% uranyl acetate, and embedded in a mixture of uranyl acetate (0.4%) and methyl cellulose (0.13%). The samples were finally subjected to observation and imaging by electron microscopy (Carl Zeiss NTS).

### Nanoparticle Tracking Analysis (NTA)

Briefly, approximately 100 µL of exosome sample was loaded into the chamber of an LM10 unit (Nanosight), and three videos of every 30 s were recorded for each sample. Data analysis was performed with NTA software (Nanosight).

### Western Blotting

Briefly, 20 μg of quantified exosomal protein was denatured by using 2 × SDS Page Buffer (Santa Cruz Biotechnology), treated at 100 °C for 5 min, separated by polyacrylamide gel electrophoresis, and transferred to membranes that were pretreated with decoloration with methanol. Blotting was performed with anti-TSG101, anti-CD9 antibody, and anti-C63 antibodies (Abcam, Cambridge, MA) and anti-cytochrome c antibody (BD Pharmingen). Antibodies against NR2B subunits of NMDAR (Upstate Biotechnology, Lake Placid, NY) and GABA_B1_R (Molecular Probes, Eugene, OR), and the GluR1 subunits of AMPAR (Chemicon, Temecula, CA) were used. Goat anti-rabbit/mouse horseradish peroxidase was used as a secondary antibody. The blots were developed with enhanced chemiluminescence (ECL) and exposed with iBright CL1000 imaging system (Invitrogen). Protein quantification was performed by bandscan and densitometry analysis with optical density for NR2B, GABAb1R, GluR1, TSG101, and CD9.

### nCounter Human miRNA Expression Assay

The nCounter human v3 miRNA expression assay designed for miRNA profiling (NanoString Technologies) was applied. The raw data (the counts for each miRNA in a sample) produced by the nCounter Digital Analyzer were subjected to technical and biological normalization using nSolver software version 2.5. DIANA-mirPath [34] was employed to perform the enrichment analysis of predicted target genes by one or more miRNAs in biological pathways.

### TaqMan miRNA Assay for Individual miRNAs

Exosomal small RNAs were isolated using the Qiagen miRNeasy Serum/Plasma Kit (Qiagen, Valencia, CA). The TaqMan miRNA Assay (Applied Biosystems, Foster City, CA) was chosen for the individual miRNA real-time PCR validation performed as the company’s protocol recommended.

### Animal Model of Post-infectious Anti-NMDAR Encephalitis

All animal procedures were approved by our Institutional Animal Care and Use Committee. Balb/c female mice, ~ 12 weeks of age, were purchased from Shanghai Laboratory Animal Center. Six mice were inoculated intranasally with HSV-1 for 2 weeks. 1 × 10^6^ plaque-forming units of HSV-1 (strain 17 syn +)^6^ were applied once daily. Blood/serum was collected at 3, 6, and 8 weeks post-inoculation and tested for anti-NMDAR antibodies through a cell-based assay as previously reported [8]. HEK293 cells transfected with subunits of NMDA receptor were fixed in 4% paraformaldehyde, permeabilized with 0.2% Triton X-100, and co-incubated with mouse sera (diluted 1:100) along with a rabbit monoclonal antibody against rabbit polyclonal antibodies against NR2B (1:200, Upstate Biotechnology) followed by the appropriate fluorescent secondary antibodies. Exosomes were isolated from pooled sera, the structures of exosomes were characterized by EM, and the concentration of exosomes was counted by NTA.

### Statistical Analysis

Data were analyzed using GraphPad Prism. One-way ANOVA followed by the Newman–Keuls post hoc test for intergroup comparisons or the nonparametric unpaired Mann–Whitney test for group comparisons was used. *p* < 0.05 was deemed statistically significant.

## Results

### Characterization of CSF Exosomes in Antibody-Positive AE Patients

**Fig. 1:**
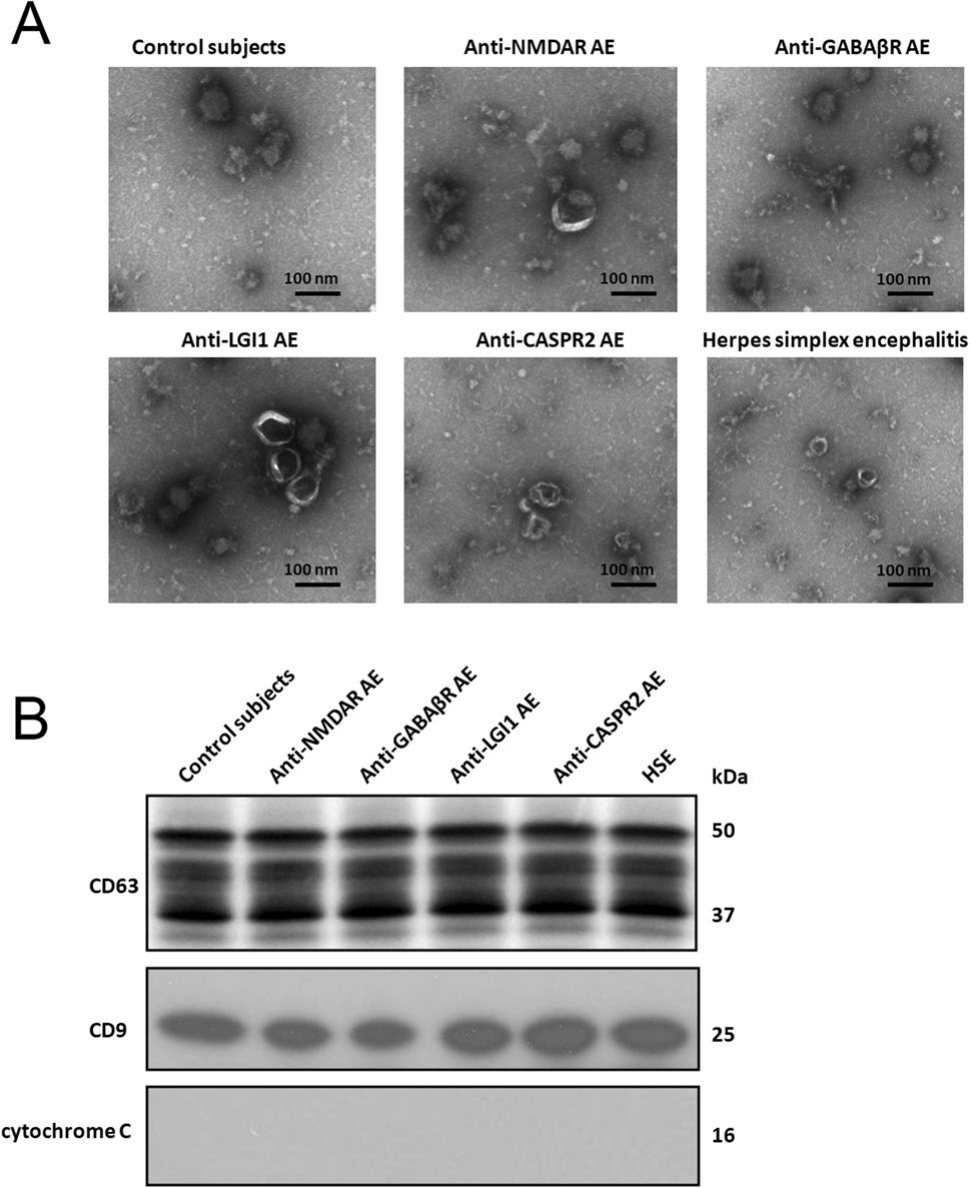
Exosome characterization. Characterization of exosome-like vesicles released from cerebrospinal fluid by **A** transmission electron microscopy of cerebrospinal fluid–derived exosomes from patients with anti-NMDA receptor encephalitis, patients with anti-GABA_B_ receptor encephalitis, patients with anti-LGI1 encephalitis, patients with anti-CASPR2 encephalitis, herpes simplex encephalitis patients, and control subjects. The scale bar indicates 100 nm. **B** Western blotting. Presence of exosomal markers, CD63 and CD9, in protein lysates from cerebrospinal fluid–derived exosomes from antibody-positive autoimmune encephalitis patients, herpes simplex encephalitis patients, and control subjects

We isolated the exosomes from CSF fluid of 13 patients with anti-NMDA receptor encephalitis, 11 patients with anti-GABA_B_ receptor encephalitis, 9 patients with anti-LGI1 encephalitis, 8 patients with anti-CASPR2 encephalitis, and 12 control individuals. Exosomes showed a spherical morphology with an average size of 94 ± 42 nm under electron microscopy (Fig. 1A). Western blot was performed using antibodies specific to CD63 and CD9. An enrichment of CD63 and CD9 was found in all exosome samples (Fig. 1B), while an absence of mitochondrial cytochrome C. These results clearly indicate that this vesicle population isolated from CSF of antibody-positive AE patients is enriched in exosomes.

### CSF-Derived Exosome miRNAs were Differentially Expressed in Antibody-Positive AE Patients

To identify the differentially expressed exosomal miRNAs derived from CSF, we performed miRNA profiling of purified exosomes isolated from CSF fluid of 13 patients with anti-NMDA receptor encephalitis, 11 patients with anti- GABA_B_ receptor encephalitis, 9 patients with anti-LGI1 encephalitis, 8 patients with anti-CASPR2 encephalitis, 9 patients with HSE, and control subjects (*n* = 12), using the NanoString Technology platform. Comparison shows that 18 miRNAs with the most significant differences in expression in AE patients with anti-NMDAR Abs compared to those of control subjects (*p* < 0.05, the unadjusted *p* value) were selected using a *t* test. Specifically, 10 miRNAs were upregulated (hsa-miR-301a-5p, hsa-miR-21-5p, hsa-miR-128-5p, hsa-miR-155-5p, hsa-miR-34a-5p, hsa-miR-326-5p, hsa-miR-132-5p, hsa-miR-29b-5p, hsa-miR-340-5p, and hsa-miR-27b-5p) and 8 downregulated (hsa-let-7d-5p, hsa-miR-15b-5p, hsa-23b-5p, hsa-miR-10a-5p, hsa-miR-146a-5p, hsa-miR-20a-5p, hsa-miR-17-5p, hsa-miR-20b-5p) in CSF-derived exosomes of AE patients (NMDAR Abs positive) versus control subjects (Table 3). These 18 exosomal microRNAs were further compared in AE patients with GABA_B_R Abs, LGI1 Ab, and CASPR2 Ab, with HSE and control subjects. These results defined a distinct signature of differential exosomal miRNAs related to the pathogenesis of antibody-positive AE. We further identified which biologic pathways were affected during the development of antibody-positive AE, we applied DIANA-mirPath on the dysregulated miRNA signature, and 48 Kyoto Encyclopedia of Genes and Genomes (KEGG) pathways were significantly enriched (*p* < 0.05; Supplementary Table 1) after false discovery rate was corrected. The results demonstrated that fatty acid biosynthesis, mucin type O-glycan biosynthesis, signaling pathways regulating pluripotency of stem cells, TGF-beta signaling pathway, Hippo signaling pathway, Wnt signaling pathway, Ras signaling pathway, and ErbB signaling pathway were significantly enriched in differentially expressed exosomic miRNAs from anti-NMDA receptor encephalitis (all *p* < 0.001). For example, the KEGG pathway “Axon guidance” (hsa04360, *p* = 0.00027; Supplementary Fig. 1) was significantly altered in differentially expressed miRNAs between anti-NMDAR encephalitis patients and control subjects at baseline, with 13 miRNAs (hsa-miR-340-5p, hsa-miR-34a-5p, hsa-miR-15b-5p, hsa-miR-17-5p, hsa-miR-20b-5p, hsa-miR-146a-5p, hsa-miR-20a-5p, hsa-miR-10a-5p, hsa-miR-155-5p, hsa-let-7d-5p, hsa-miR-21-5p, hsa-miR-301a-5p, hsa-miR-27b-5p) targeting 56 genes, including EFNB2, GSK3B, MET, ROCK1, NRAS, PAK2, NGEF, and ARHGEF12. These results demonstrated that anti-NMDAR encephalitis associated miRNAs could dysregulate key cellular signal pathways, further may predispose AE.

**Table 3:** miRNAs upregulated and downregulated in antibody-positive autoimmune encephalitis subtypes

miRNA ID	Counts in NMDAR group	Counts in control group	Fold change (95%CI)	*p* value	Counts in GABAβR Group	Fold change (95%CI)	*p* value	Counts in LGI1 group	Fold change (95%CI)	*p* value	Counts in CASPR2 group	Fold change (95%CI)	*p* value	Counts in HSE Group	Fold change (95%CI)	*p* value
hsa-miR-301a-5p	221.58 ± 27.89	21.58 ± 3.58	**10.997 (6.391–13.225)**	0.014	45.33 ± 10.04	**2.114(1.002–3.279)**	0.021	77.68 ± 15.55	**3.658(2.687–5.074)**	0.173	210.73 ± 25.86	**9.928(7.312–12.354)**	0.002	25.36 ± 4.55	**1.175(0.859–1.521)**	0.228
hsa-miR-21-5p	231 ± 20.87	27.23 ± 7.94	**8.387(5.063–11.118)**	0.034	94.35 ± 18.73	**3.417(2.016–3.214)**	0.034	32.06 ± 6.56	**1.186(0.759–1.689)**	0.611	105.44 ± 29.52	**4.646(3.013–6.041)**	0.001	30.36 ± 8.56	**1.115(0.841–1.355)**	0.556
hsa-miR-128-5p	93.05 ± 11.70	14.25 ± 2.06	**7.876(5.138–10.242)**	0.020	51.63 ± 14.48	**3.567(1.087–5.045)**	0.036	135.10 ± 19.33	**9.47(7.146–11.287)**	0.021	4.09 ± 1.28	*0.295(0.137–0.412)*	0.021	18.56 ± 3.33	**1.302(1.025–1.639)**	0.057
hsa-miR-155-5p	325 ± 39.19	47.08 ± 8.17	**6.616(4.089–9.411)**	0.040	248.62 ± 33.22	**5.287(3.416–7.102)**	0.053	19.11 ± 5.27	**0.417(0.189–0.682)**	0.505	465.33 ± 121.23	**9.529(7.513–12.347)**	0.023	56.87 ± 10.23	**1.208(0.987–1.553)**	0.126
hsa-miR-34a-5p	146.77 ± 42.10	27.77 ± 5.99	**5.577(3.093–8.084)**	0.009	116.55 ± 25.14	**4.208(3.091–6.156)**	0.042	235.47 ± 89.24	**4.623(2.986–6.354)**	0.195	281.37 ± 46.57	**10.575(8.541–13.245)**	0.003	36.75 ± 7.44	**1.323(0.997–1.753)**	0.432
hsa-miR-326-5p	105.36 ± 11.58	20.18 ± 4.58	**4.986(2.346–6.117)**	0.011	193.22 ± 45.17	**9.721(7.057–11.751)**	0.097	85.21 ± 11.13	**4.359(2.986–5.894)**	0.253	14.23 ± 5.89	*0.629(0.421–0.911)*	0.265	30.54 ± 5.97	**1.513(1.089–1.917)**	0.032
hsa-miR-132-5p	44.58 ± 8.30	10.25 ± 5.26	**3.526(2.200–5.007)**	0.020	97.15 ± 10.48	**9.537(7.024–11.762)**	0.095	90.41 ± 24.83	**8.565(7.011–10.547)**	0.000	76.01 ± 14.68	**6.811(5.017–8.412)**	0.004	8.55 ± 2.12	*0.834(0.611–1.013)*	0.551
hsa-miR-29b-5p	61.58 ± 9.45	28.05 ± 5.62	**2.196(1.287–3.042)**	0.020	94.42 ± 25.83	**3.488(1.341–5.172)**	0.035	208.36 ± 70.77	**7.52(6.342–8.024)**	0.550	177.30 ± 36.45	**6.516(5.023–8.151)**	0.013	25.56 ± 4.11	*0.911(0.751–1.059)*	0.178
hsa-miR-340-5p	110.47 ± 14.95	60.22 ± 9.49	**1.827(0.927–3.041)**	0.012	356.86 ± 51.95	**5.947(4.159–6.853)**	0.060	248.78 ± 51.82	**4.032(2.698–5.898)**	0.514	19.09 ± 7.93	*0.302(0.112–0.511)*	0.007	70.54 ± 11.54	**1.171(0.895–1.425)**	0.522
hsa-miR-27b-5p	62.16 ± 7.80	43.25 ± 9.18	**1.436(0.732–2.039)**	0.020	35.11 ± 7.51	*0.6(0.343–0.956)*	0.206	298.50 ± 36.06	**7.661(5.995–8.942)**	0.002	25.37 ± 7.39	*0.594(0.405–0.714)*	0.017	59.45 ± 10.27	**1.374(1.024–1.785)**	0.054
hsa-let-7d-5p	81.33 ± 12.72	132.76 ± 20.68	*0.62(0.311–1.184)*	0.012	40.81 ± 9.60	*0.295(0.002–0.461)*	0.073	31.42 ± 8.24	*0.188(0.042–0.287)*	0.075	130.77 ± 27.43	*0.999(0.741–1.245)*	0.854	68.23 ± 10.74	*0.514(0.214–0.714)*	0.031
hsa-miR-15b-5p	51.29 ± 10.53	108.56 ± 31.33	*0.474(0.155–0.844)*	0.013	354.31 ± 41.63	**3.4(1.642–5.317)**	0.034	65.14 ± 9.42	*0.588(0.237–0.724)*	0.017	162.32 ± 25.12	**1.553(1.142–1.872)**	0.201	97.52 ± 21.54	*0.898(0.714–1.157)*	0.558
hsa-miR-23b-5p	39.20 ± 7.28	133.35 ± 18.21	*0.294(0.06–0.596)*	0.002	94.64 ± 17.57	*0.719(0.314–1.023)*	0.087	94.51 ± 27.48	*0.682(0.428–0.855)*	0.022	51.18 ± 9.63	*0.423(0.215–0.711)*	0.024	100.27 ± 15.21	*0.751(0.539–0.974)*	0.609
hsa-miR-10a-5p	25.10 ± 3.24	109.65 ± 26.63	*0.229(0.049–0.479)*	0.002	683.99 ± 95.09	**5.92(4.145–7.245)**	0.059	35.45 ± 9.49	*0.226(0.024–0.405)*	0.004	36.09 ± 6.24	*0.329(0.157–0.503)*	0.063	98.54 ± 15.24	*0.898(0.642–1.224)*	0.430
hsa-miR-146a-5p	66.35 ± 8.63	403 ± 57.62	*0.164(0.004–0.279)*	0.020	201.42 ± 74.92	*0.486(0.123–0.841)*	0.305	361.06 ± 58.67	*0.858(0.655–1.056)*	0.230	361.63 ± 52.02	*0.872(0.701–1.051)*	0.789	355.21 ± 44.17	*0.880(0.678–1.057)*	0.237
hsa-miR-20a-5p	104.72 ± 15.90	677.62 ± 78.66	*0.155(0.004–0.294)*	0.019	601.80 ± 98.28	*0.915(0.566–1.345)*	0.009	4.82 ± 1.46	*0.006(0–0.019)*	0.004	541.61 ± 163.12	*0.86(0.674–1.019)*	0.587	547.25 ± 65.89	*0.807(0.614–1.051)*	0.358
hsa-miR-17-5p	93.66 ± 20.57	633.91 ± 70.95	*0.147(0.003–0.381)*	0.014	59.75 ± 7.26	*0.085(0.001–1.187)*	0.059	565.65 ± 140.96	*0.842(0.697–1.120)*	0.045	497.49 ± 82.34	*0.745(0.598–0.914)*	0.644	532.37 ± 52.38	*0.839(0.689–1.041)*	0.657
hsa-miR-20b-5p	84.01 ± 26.62	603.07 ± 75.71	*0.136(0.001–0.269)*	0.019	595.74 ± 95.12	*0.989(0.489–1.489)*	0.113	358.41 ± 39.89	*0.602(0.432–0.896)*	0.000	132.40 ± 37.32	*0.166(0.032–0.305)*	0.155	421.57 ± 41.87	*0.699(0.435–0.987)*	0.276

The *p* value describes significance of difference between AE subtype in comparison to healthy controls using nSolver Software. *miRNA ID*, official microRNA name according to miRBASE. *p* value = the unadjusted *p* value from the *t* test. Bold values represent higher expression; italicized values represent lower expression

### Cancer-Associated Pathways Enriched in AE Dysregulated miRNAs

**Fig. 2:**
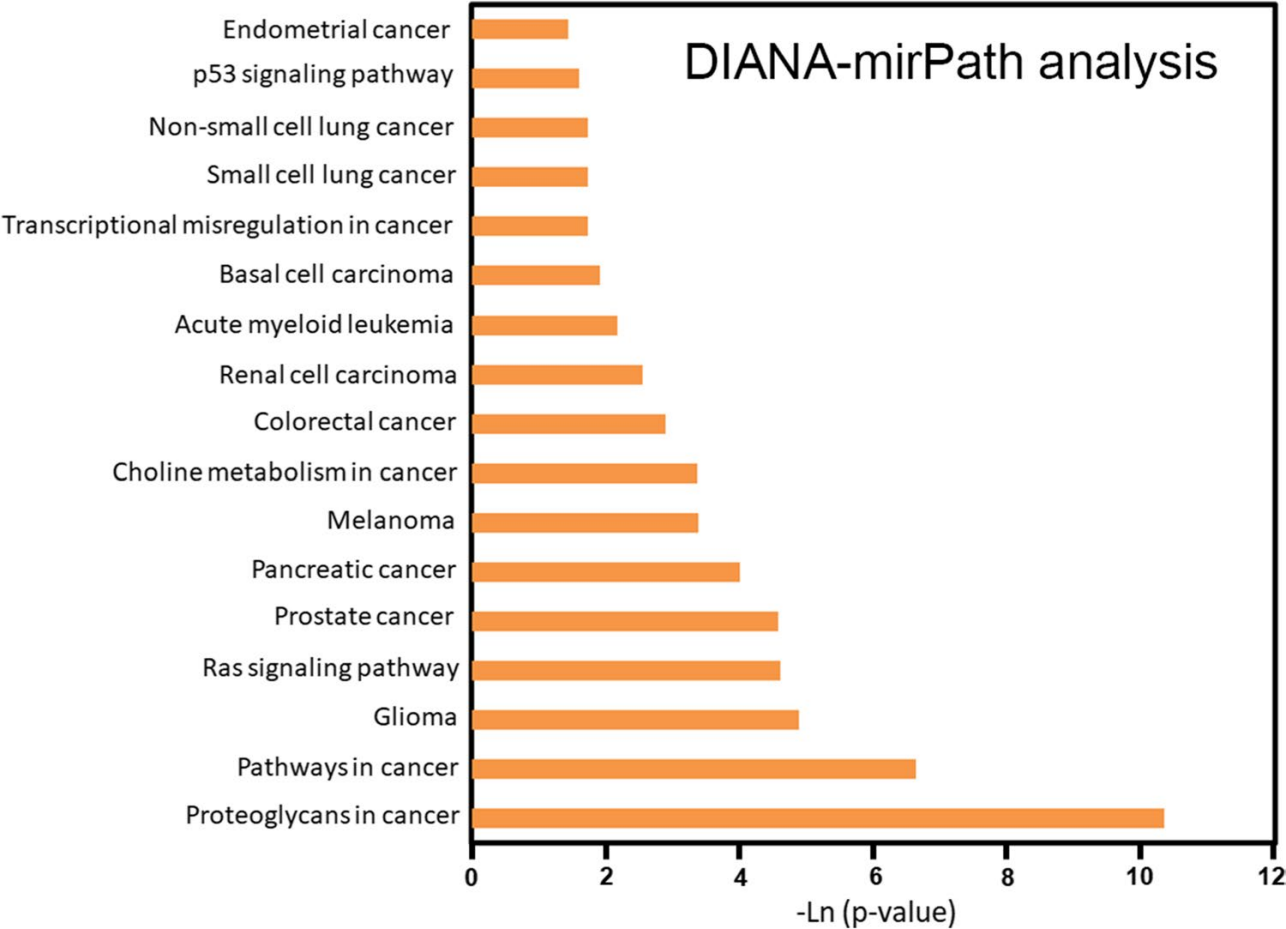
Cancer-associated Kyoto Encyclopedia of Genes and Genomes (KEGG) pathways enriched in anti-NMDAR autoimmune encephalitis-associated dysregulated miRNAs. Graphical representation of anti-NMDAR‐mediated targeted pathways generated using DIANA‐miRPath (*y*‐axis: *p* values; *x*‐axis: miRNA‐associated pathways). *p* values for pathway analysis were obtained by Fisher’s exact test as enrichment analysis method and the false discovery rate (FDR) was estimated using the Benjamini and Hochberg method

The presence of an occult tumor was found in the AE patients that serves as a stimulus for autoantibody production [35, 36]. Interestingly, there were 17 cancer-associated pathways enriched into the dysregulated 18 miRNAs, including endometrial cancer, p53 signaling pathway, non-small cell lung cancer, small cell lung cancer, transcriptional misregulation in cancer, basal cell carcinoma, acute myeloid leukemia, renal cell carcinoma, colorectal cancer, choline metabolism in cancer, melanoma, pancreatic cancer, prostate cancer, Ras signaling pathway, glioma, pathways in cancer, and proteoglycans in cancer (*p* < 0.02; Fig. 2). For example, the KEGG pathway “Pathways in cancer” (hsa05200, *p* = 2.312e − 07, Supplementary Fig. 2) was significantly altered in differentially expressed miRNAs between anti-NMDAR encephalitis patients and control subjects at baseline, with 15 miRNAs (hsa-miR-20a-5p, hsa-miR-17-5p, hsa-miR-20b-5p, hsa-miR-340-5p, hsa-miR-21-5p, hsa-let-7d-5p, hsa-miR-15b-5p, hsa-miR-155-5p, hsa-miR-27b-5p, hsa-miR-34a-5p, hsa-miR-146a-5p, hsa-miR-10a-5p, hsa-miR-301a-5p, hsa-miR-132-5p, hsa-miR-23b-5p) targeting 160 genes, including FZD7, BRAF, FGF12, FOS, GSK3B, PRKCA, STAT3, PDGFRA, FZD5, and E2F1. Data from these experiments demonstrated that exosomes expressing specific miRNAs may participate in cancer development, which indicates a feedback regulation from antibody-positive AE.

### Validation of miRNA Expression Using Independent Cohorts of Antibody-Positive AE Patients

**Fig. 3:**
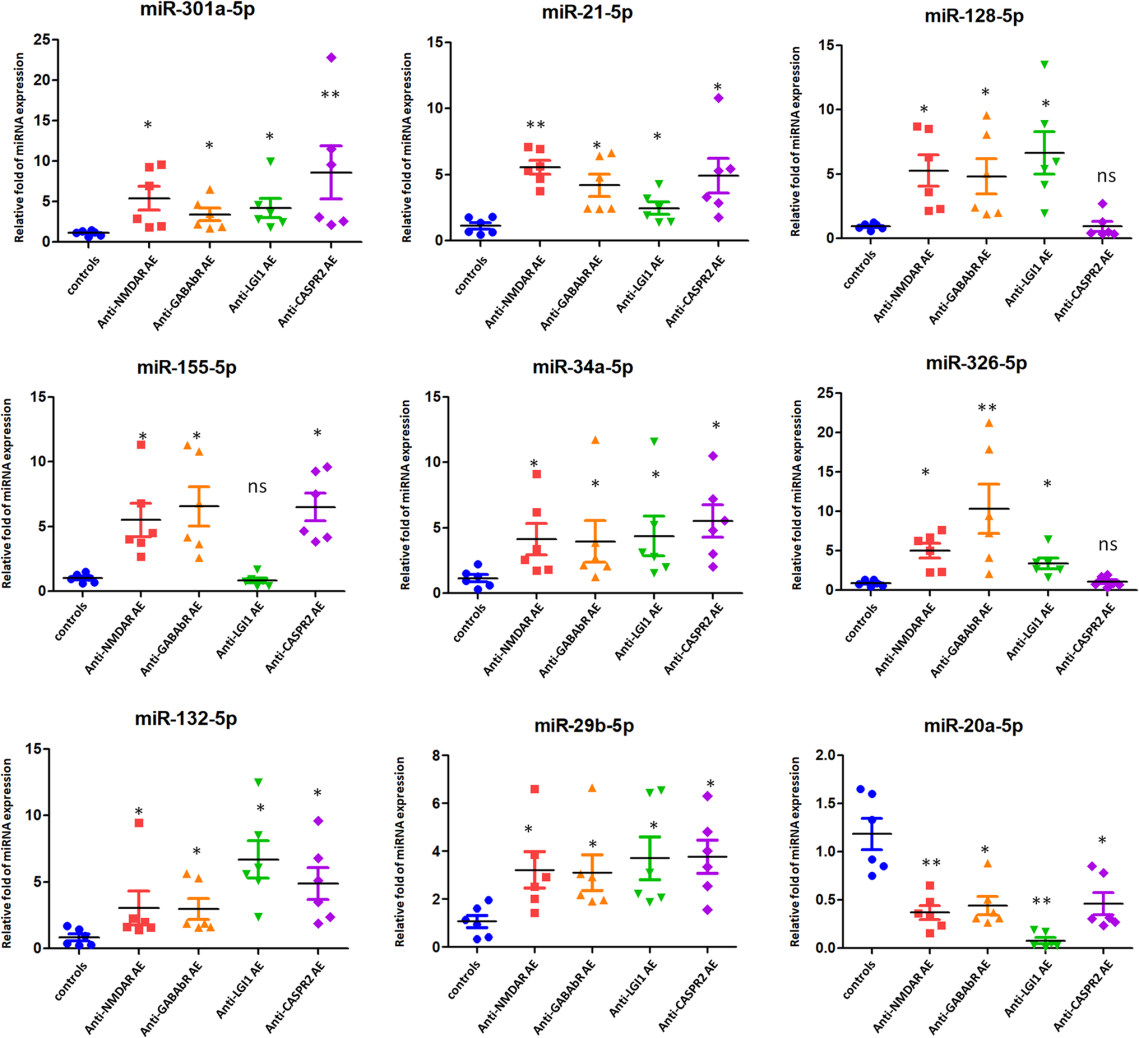
Validation of miRNA expression. TaqMan real-time PCR to validate the expression levels of nine miRNAs (miR-301a-5p, miR-21-5p, miR-128-5p, miR-155-5p, miR-34a-5p, miR-326-5p, miR-132-5p, miR-29b-5p, and miR-20a-5p) selected for further validation using an independent cohort of 6 patients with anti-NMDA receptor encephalitis, 6 patients with anti-GABA receptor encephalitis, 6 patients with anti-LGI1 encephalitis, 6 patients with anti-CASPR2 encephalitis, and 6 control individuals. Data shown are as mean ± SEM. *p < 0.05; **p < 0.01; ***p < 0.001

The miRNAs were selected for validation since they were found to relate with neuroinflammation and encephalitis [37–42]. Nine miRNAs (miR-301a-5p, miR-21-5p, miR-128-5p, miR-155-5p, miR-34a-5p, miR-326-5p, miR-132-5p, miR-29b-5p, and miR-20a-5p) were selected for further validation using an independent cohort of 6 patients with anti-NMDA receptor encephalitis, 6 patients with anti-GABA_B_ receptor encephalitis, 6 patients with anti-LGI1 encephalitis, 6 patients with anti-CASPR2 encephalitis, and 6 control individuals. We found that miR-301a-5p, miR-21-5p, miR-34a-5p, miR-132-5p, and miR-29b-5p were significantly highly expressed in CSF exosomes in anti-NMDAR, GABA_B_R, LGI1, and CASPR2 AE patients when compared with healthy controls (*p* < 0.05), while miR-20a-5p was significantly lowly expressed in CSF exosomes (Fig. 3). In addition, we investigated another three miRNAs (miR-128-5p, miR-155-5p, and miR-326-5p) using individual CSF exosomes. miR-128-5p and miR-326-5p were significantly increased in CSF exosomes isolated from anti-NMDAR, GABA_B_R, and LGI1 AE patients when compared with healthy controls (*p* < 0.05); however, there was no significant difference of these two miRNAs between anti-CASPR2 AE patients and healthy controls (Fig. 3). Exosomal miR-155-5p was significantly highly expressed in anti-NMDAR, GABA_B_R, and CASPR2 AE patients when compared with healthy controls (*p* < 0.05); however, there was no significant difference of exosomal miR-155-5p between anti-LGI1 AE patients and healthy controls (Fig. 3). Taken together, these data confirmed the validity of differentially expressed exosomal miRNAs in CSF related to the pathogenesis of antibody-positive AE.

### Exosome Concentration Was Higher in HSE Patients Compared to HSV-Negative Patients

**Fig. 4:**
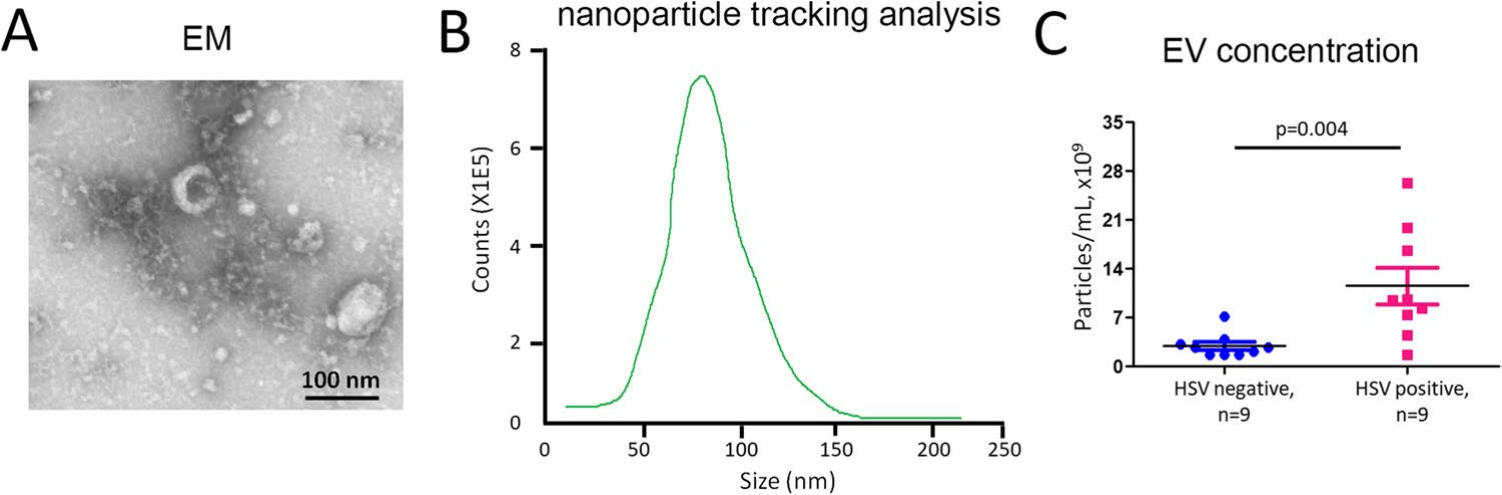
Exosome was induced for secretion into CSF in HSE compared to HSV( −) patients. **A** The electron micrographs of the microvesicles. **B** Microvesicle concentration was measured by nanoparticle tracking analysis (NTA). **C** Comparison of exosome concentration between 9 HSE patients compared to 9 HSV( −) patients

In order to determine whether the abundance of exosomes is changed in HSE and to define their compositions and roles, exosomes were isolated from CSF from 9 HSE patients and 9 HSV( −) patients. Exosomic structure was examined by TEM, and exosomic concentration was measured by NTA. The electron micrographs of the microvesicles revealed rounded structures with a size of 85–120 nm (Fig. 4A), similar to previously described exosomes. A similar size distribution was also confirmed by NTA with a peak around 93 nm (Fig. 4B). We sought to determine whether there were differences in the abundance of exosome production isolated from CSF in HSE patients compared to HSV( −) patients. An analysis of a cross-sectional cohort revealed a significantly higher EV concentration in 9 HSE patients compared to 9 HSV( −) patients (HSV( +): 11.65 × 10^9^ ± 2.33 × 10^9^ vs. HSV( −): 3.81 × 10^9^ ± 0.54 × 10^9^ particles/mL; *p* = 0.004; Fig. 4C). These data indicated that HSV infection would increase the production of exosomes secreted into CSF in HSE, suggesting a potential role of exosomes in HSE.

### Exosome Was Induced in Mouse Model of HSE

**Fig. 5:**
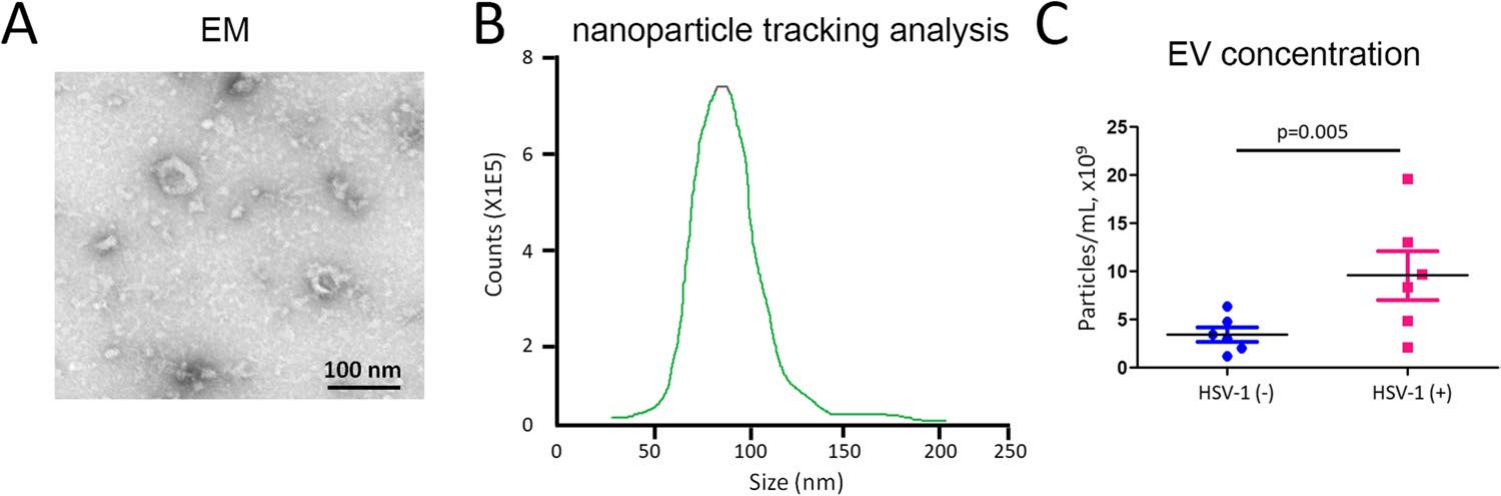
Exosome was induced in mouse model of HSE. **A** The electron micrographs of the exosomes derived from sera of mouse model of HSE. **B** Nanoparticle tracking analysis (NTA) to show size distribution of exosomes derived from sera of mouse model of herpes simplex encephalitis. **C** Comparison of the difference of exosome concentration in HSV-treated mice (*n* = 6) compared to control group (*n* = 6)

In order to validate the results obtained from clinical subjects, we developed an endogenous rodent model of post-infectious encephalitis. Six mice were inoculated intranasally with HSV-1. Another six age- and sex-matched control Balb/c mice were inoculated with vehicle solution. Serum was collected at 8 weeks post-inoculation and tested for exosome content. The electron micrographs of the exosomes revealed rounded structures with a size of 65–94 nm (Fig. 5A). NTA demonstrated a similar size distribution with a peak around 90 nm (Fig. 5B). We compared the exosome production between HSV-treated mice and control group. Results revealed a significantly higher exosome concentration in HSV-treated mice (*n* = 6) compared to the control group (*n* = 6) (HSV-1( +): 10.22 × 10^9^ ± 2.19 × 10^9^ vs. HSV-1( −): 4.55 × 10^9^ ± 0.33 × 10^9^ particles/mL; *p* = 0.005; Fig. 5C). These data confirmed that exosomes were induced for secretion after HSV infection, providing more evidence of the pathogenetic role of exosomes in HSE.

### Exosomes Isolated from HSE Patients Expressed Higher NMDAR, GABAR, and AMPAR Subunits

**Fig. 6:**
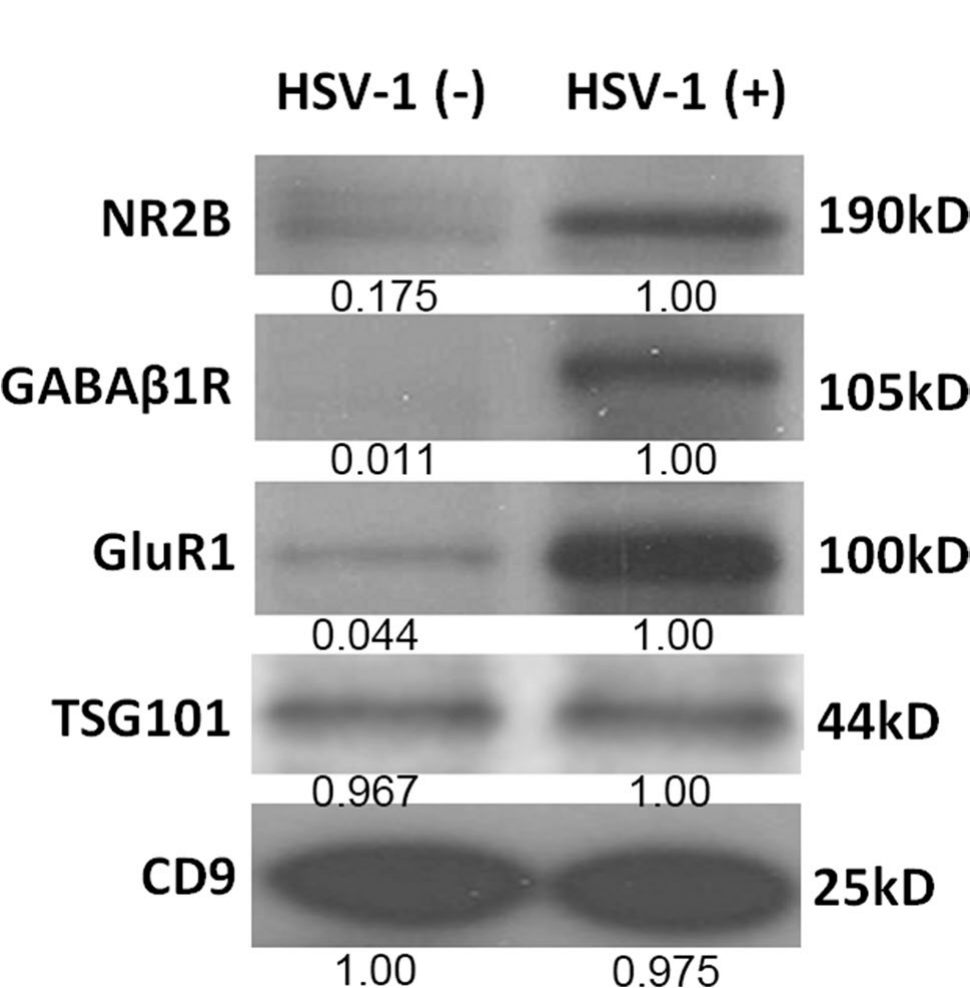
Exosome expressed higher NMDAR, GABAR, and AMPAR subunits in HSV-induced animal model. Western blot to show the protein level of NR2B subunits of NMDAR and GABA_B1_R, and the GluR1 subunits of AMPAR on the pooled sera exosomes from 6 HSV-treated mice compared to those in control group (*n* = 6). Protein quantification was performed by bandscan and densitometry analysis with optical density for NR2B, GABAb1R, GluR1, TSG101, and CD9. TSG101 and CD9 were detected as the exosome markers and loading control

We next examine the exosomal protein composition, especially neuronal autoantigens. Exosomes isolated from pooled sera collected from 6 HSV-treated mice were subjected for western blot, and results demonstrated that NR2B subunits of NMDAR and GABA_B1_R, and the GluR1 subunits of AMPAR were present and highly expressed in HSV-1-treated mice compared to those in control group (Fig. 6). TSG101 and CD9 were detected as the exosome markers and loading control.

### miR-H2-3p and miR-H4-3p Encoded by HSV Were Dysregulated in Exosomes Isolated from CSF of HSE Patients

**Fig. 7:**
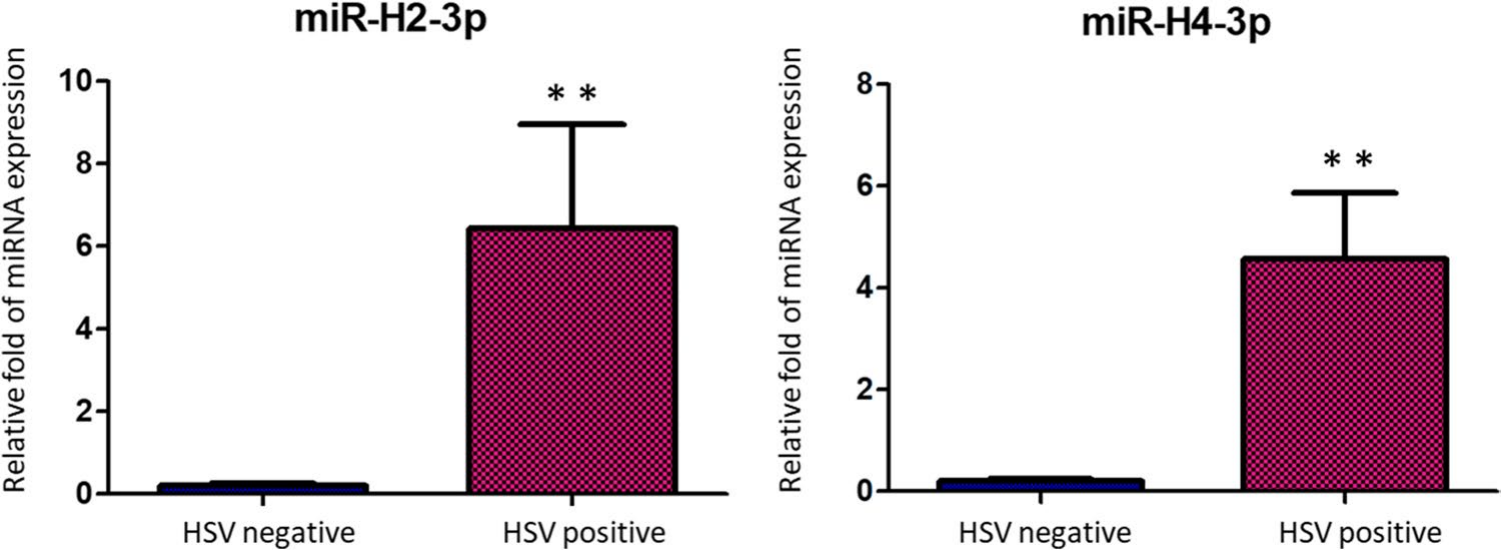
Exosomal miR-H2-3p and miR-H4-3p miRNAs expressed by HSV. miR-H2-3p and miR-H4-3p were compared for expression from cerebrospinal fluid exosomes isolated from 5 HSE patients compared to 5 HSV( −) patients. Relative expression of miR-H2-3p and miR-H4-3p was calculated and observed statistically differentially expressed compared to the control group. *p < 0.05, **p < 0.01

We further examined the presence of miRNAs encoded and expressed by HSV in exosomes isolated from CSF of patients with HSE. miR-H2-3p, miR-H4-3p, miR-H3, miR-H4-5p, miR-H5, and miR-H6 were chosen for TaqMan microRNA detection assay. Only miR-H2-3p and miR-H4-3p were detectable in CSF exosomes from HSE. However, miR-H3, miR-H4-5p, miR-H5, and miR-H6 were not detectable in both groups. After comparison, we found that miR-H2-3p and miR-H4-3p were significantly highly expressed in CSF-derived exosomes isolated from HSE patients (*n* = 5) when compared to those in HSV( −) subjects (*n* = 5) (miR-H2-3p, *p* < 0.01; miR-H4-3p, *p* < 0.01; Fig. 7). These data suggested that HSV-encoded miRNAs may potentially target host genes to regulate HSE by the way of exosomal genetic transfer.

## Discussion

Concurrent inflammatory findings in the CSF, including the presence of OCBs, lymphocytic pleocytosis, and elevated protein levels, were found in antibody-mediated AE [43]. In this study, we presented the evidence to support the role of exosomes and exosomal expression of miRNAs and neuronal antigens to the pathophysiology of HSE and AE. Results from the current study demonstrated dysregulated miRNAs in CSF-derived exosomes of AE patients (NMDAR Abs positive) versus control subjects. Similar expression patterns were demonstrated in AE patients with anti-GABA_B_R Abs, anti-LGI1 Abs, and anti-CASPR2 Abs (Table 3). Further study demonstrated important enriched cancer-associated pathways related to the dysregulated miRNAs, including endometrial cancer, the p53 signaling pathway, non-small cell lung cancer, small cell lung cancer, transcriptional dysregulation in cancer, basal cell carcinoma, acute myeloid leukemia, renal cell carcinoma, colorectal cancer, choline metabolism in cancer, melanoma, pancreatic cancer, prostate cancer, the Ras signaling pathway, glioma, pathways in cancer, and proteoglycans in cancer (Fig. 2). These results indicated that exosomes expressing specific miRNAs may participate in cancer development, which indicates a feedback regulation from antibody-positive AE. Ectopic expression of neuronal antigens in various types of malignant tumors could trigger the anti-tumor immune response cross-reacting with the cognate CNS protein, which often leads to the paraneoplastic neurological syndromes [44].

MicroRNAs are a group of short, noncoding RNA molecules that post-transcriptionally regulate the expression of target mRNAs. Inflammatory processes may be promoted by miRNAs [45], such as miR-155, or suppressed by miRNAs, including miR-146a, miR-124, and miR-21. miR-155 is a central proinflammatory mediator of the CNS in response to TLR signaling [46–48]. miR-155 targets include SOCS1 [48], SHIP1 [49], C/EBP-β [50], and IL13Rα1 [51]. In addition, miR-155 expression is induced by p53 in immune cells, and miR-155 subsequently targets c-Maf, which induces differentiation and anti-inflammatory responses [52]. In our study, we found that miR-155 was significantly more highly expressed in anti-NMDAR, GABA_B_R, and CASPR2 AE patients than in control subjects (*p* < 0.05; Fig. 3). These data demonstrated that miR-155 may participate in autoimmune encephalitis by regulating the SOCS1, SHIP1, C/EBP-β, and IL13Rα1 signaling cascades in microglia. Anti-voltage-gated potassium channel (VGKC) antibodies include antibodies against associated proteins LGI1 and CASPR2 in limbic encephalitis [53]. Results in our study demonstrated that miRNAs 128-5p was found to be over-expressed in CASPR2 and LGI1 AE cases, and miR-155-5p was found highly expressed in CASPR2 encephalitis, while not in LGI1 encephalitis (Fig. 3). These data indicated that both miRNAs 128-5p and 155-5p participated in the encephalitis caused by anti-VGKC antibodies. Pathways associated with SOCS1, SHIP1, C/EBP-β, and IL13Rα1 signaling cascades might regulate the development CASPR2 encephalitis but not LGI1 encephalitis. miR-128 was reported to participate in the inflammation pathogenesis by regulating the p38α/M-CSF inflammatory signaling pathway [54]. Thus, p38α/M-CSF inflammatory signaling pathway might fine-tune anti-LGI1- and anti-CASPR2-associated encephalitis. Furthermore, p53 also suppresses c-Maf through the induction of two additional microRNAs, miR-34a and miR-145, which target Twist2, an activator of c-Maf expression [52]. The results of our study demonstrated that miR-34a-5p was significantly more highly expressed in CSF exosomes in anti-NMDAR, GABA_B_R, LGI1, and CASPR2 AE patients than in control subjects (*p* < 0.05; Fig. 3), which indicated that miR-34a could inhibit the anti-inflammatory effect in AE by regulating Twist2 mRNA. Additionally, we found that miR-301a-5p was significantly more highly expressed in CSF exosomes in anti-NMDAR, GABA_B_R, LGI1, and CASPR2 AE patients than in control subjects. There are several potential mechanisms underlying the role of miR-301a in the development of AE. Bibhabasu Hazra et al. [55] reported that the expression of miR-301a is increased in Japanese encephalitis virus (JEV)–infected microglial cells and the human brain. Overexpression of miR-301a augments the JEV-induced inflammatory response, whereas inhibition of miR-301a completely reverses the effects. They further found that miR-301a regulates the inflammatory response to JEV infection via suppression of NKRF activity [55]. We have not assessed the effect on the targeted exosomal miRNA pathways in the study. Future functional study on animal model of AE should be employed by targeting the specific miRNAs or its regulated pathways.

Infection with HSV leads to blisters or sores in the epithelia, and HSV further survives in sensory neurons via a life-long latent reservoir [56]. Although asymptomatic pathology usually occurs after infection with HSV, periodic reactivation of HSV within latently infected neurons may lead to recurrent infections [57]. HSV is an important cause of encephalitis, which is the most common type of sporadic encephalitis in humans, with a high mortality rate of up to 70% if left untreated [13]. HSE has common and even severe symptoms, including fever, headaches, vomiting, neurological deficits, and seizures [58]. Recent studies have identified the specific types of immune cells that induce brain inflammation in HSE [59]. Using a mouse model, the authors showed that neutrophils increased the permeability of the blood–brain barrier and further led to brain damage, which is commonly found in HSE [59]. At the same time, monocytes were found to play a protective role by clearing HSV and limiting viral replication, previously evidenced as an important way to prevent brain damage [59]. Therefore, immune cells infiltrate the CNS, and disruption of the blood–brain barrier causes a neuroinflammatory response during HSE [60]. However, the mechanisms by which HSV causes encephalitis remain poorly understood. The results of this study demonstrated that exosomes were induced for secretion into local brain sites and even into sera in HSE (Fig. 4). Viral infections are considered one more trigger of antibody-mediated encephalitis [61]. NMDAR autoantibodies in patients were firstly discovered in patients with HSE, which was leading to the hypothesis that autoimmune-mediated clinical symptoms happened after HSV infection [15]. Further studies validated that there was a frequency of almost 1/3 of patients with HSE developing NMDAR encephalitis thereafter [14, 16]. In addition, HSV-induced exosome production was validated in an animal model of post-HSV encephalitis (Fig. 5). More importantly, exosomes isolated from pooled sera collected from 6 HSV-treated mice demonstrated that NR2B subunits of NMDAR and GABA_B1_R, and the GluR1 subunits of AMPAR were present and highly expressed in HSV-1-treated mice (Fig. 6). These data indicated that exosomes play critical roles in the presentation of surface autoantigens, inducing autoimmunity and inflammation, which could be recognized as a novel mechanism for HSE pathogenesis. Due to the challenge in the collection of sufficient CSF samples from HSE patients, exosomes isolated from the CSF of HSV-positive patients are required to compare the protein expression levels of NMDAR, GABAR, and AMPAR subunits from those of HSV( −) patients. It was reported that experimental mice inoculated intranasally with HSV-1 developed serum NMDAR antibodies [17]. Autoimmunity also leads to development of autoantibodies to GABAa, AMPA, and dopamine D2 receptors [62]. The type of autoimmunity effect extended to other viruses, such as Epstein-Barr virus, varicella-zoster virus, herpesvirus, hepatitis viruses, and Japanese encephalitis B virus, which indicated that virus-induced autoantibody generation could be a broad mechanism of pathology in autoimmune neurological disease [63]. However, it requires more investigation to prove the causal relationship.

The only abundant viral gene product from HSV-1 after it established latency in neurons is the latency-associated transcript (LAT), which has been proven to be a noncoding RNA [12, 64]. Umbach et al. reported that LAT encoded four distinct miRNAs. Among them, miR-H2-3p was transcribed complementarily to mRNA of infected cell protein 0 (ICP0), a viral immediate-early transcriptional activator in HSV-1 replication and latency [65]. A fifth HSV-1 miRNA, miR-H6, was identified in latently infected trigeminal ganglia [66]. miR-H6 inhibits ICP4 mRNA translation by seeding region complementarity to the target mRNA; thus, miR-H6 is required for HSV-1 productive infection [66]. Latency HSV-encoded miRNAs, miR-H2-3p and miR-H4-3p, coordinate with human miR-138, which is highly abundant in neurons, to promote latency during lytic infection [67, 68]. The results from our current study demonstrated that miR-H2-3p and miR-H4-3p were significantly more highly expressed in CSF-derived exosomes isolated from HSE patients than those isolated from HSV( −) subjects (miR-H2-3p, *p* < 0.01; miR-H4-3p, *p* < 0.01; Fig. 7). Therefore, exosomal expression of miR-H2-3p may further inhibit ICP0 protein expression, thus decreasing HSV entry into the productive replication cycle and latency [65, 69, 70], which may be further considered a potential mechanism for HSE in patients. Increased exosomal miR-H4-3p expression may inhibit ICP34.5 expression via an “antisense” mechanism [71, 72]. In addition, we found that miR-H3, miR-H4-5p, miR-H5, and miR-H6 were not detectable in exosomes isolated from the CSF of all patients, indicating that these viral miRNAs could not be taken up by exosomes shed by brain cells.

There are several limitations in this study. First, caution should be taken to understand the results because of the relatively small sample size in the current study. A larger cohort study for validation will be required in the future. Second, abolishment of exosome secretion induced by HSV is needed to confirm its in vivo role in HSE. The detailed molecular mechanisms underlying HSV-induced exosomes during encephalitis require further investigation.

In conclusion, our results demonstrated that there was a specific miRNA signature that was differentially expressed in antibody-positive AE patients. Functionally, the dysregulated miRNAs were enriched in cancer-associated pathways, indicating there was a feedback regulation of AE in cancer development. Exosomes could be induced during HSV infection. These secreted exosomes contained higher expression of NMDAR, GABAR, and AMPAR subunits as well as miR-H2-3p and miR-H4-3p encoded by HSV in encephalitis patients as well as HSV-induced animal model. Therefore, we concluded that HSV-induced exosomes are critical players in HSE development via the presentation of surface/cellular neuronal autoantigens that trigger brain autoimmunity. These findings provide potential novel strategies for therapeutic intervention.

## Supplementary Information

Below is the link to the electronic supplementary material.

Supplementary file1 (TIF 6299 KB)

Supplementary file2 (TIF 21291 KB)

Supplementary file3 (DOCX 22 KB)

Supplementary file4 (DOCX 23 KB)

## Data Availability

The datasets generated and/or analyzed during the current study are available from the corresponding author on reasonable request. The study was approved by the institutional ethics committee of Sir Run Run Shaw Hospital affiliated with Zhejiang University School of Medicine, and the need for informed consent was received from all subjects. The study was performed in accordance with the 1964 Declaration of Helsinki and its later amendments. All animal procedures were approved by our Institutional Animal Care and Use Committee at Sir Run Run Shaw Hospital affiliated with Zhejiang University.

## Abbreviations

HSE: Herpes simplex encephalitis
AE: Autoimmune encephalitis
CSF: Cerebrospinal fluid
NMDAR: *N*-Methyl-d-aspartate receptor
GABA_B_R: Gamma-aminobutyric acid-B receptor
LGI: Leucine-rich glioma-inactivated 1
CASPR2: Contactin-associated protein-like 2 receptor
ANNA-1: Anti-neuronal nuclear antibody type 1
CRMP5: Collapsin-responsive mediator protein-5
