# Supplementary figures and images for: Cerebrospinal Fluid Extracellular Vesicles with Distinct Properties in Autoimmune Encephalitis and Herpes Simplex Encephalitis
Source: Mol Neurobiol. 2022 Jan 27;59(4):2441–55. doi: 10.1007/s12035-021-02705-2 (PMC9016041; doi:10.1007/s12035-021-02705-2)

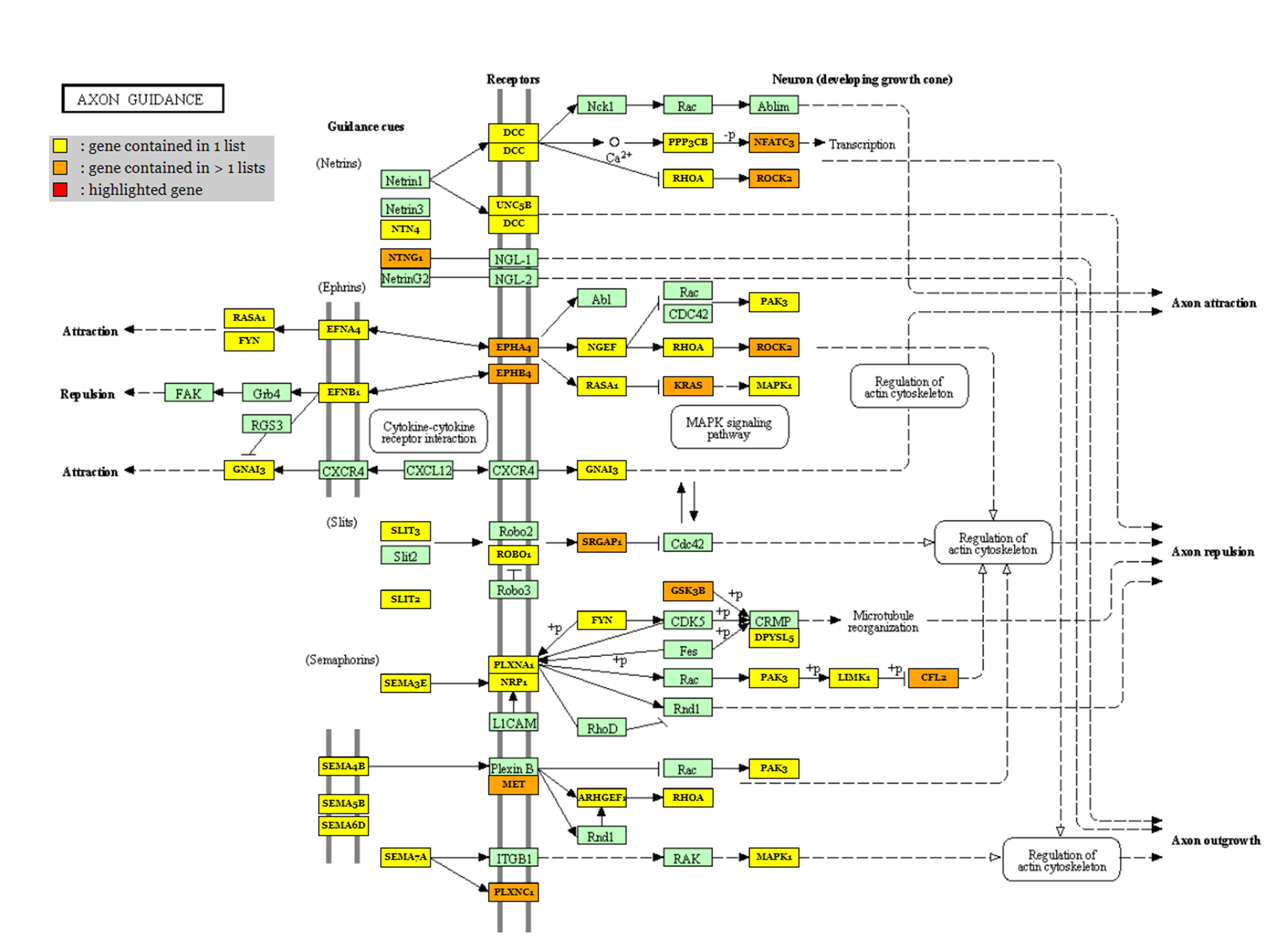

Supplement: Supplementary file 1 — Supplementary file1 (TIF 6299 KB) [file 12035_2021_2705_MOESM1_ESM.tif]

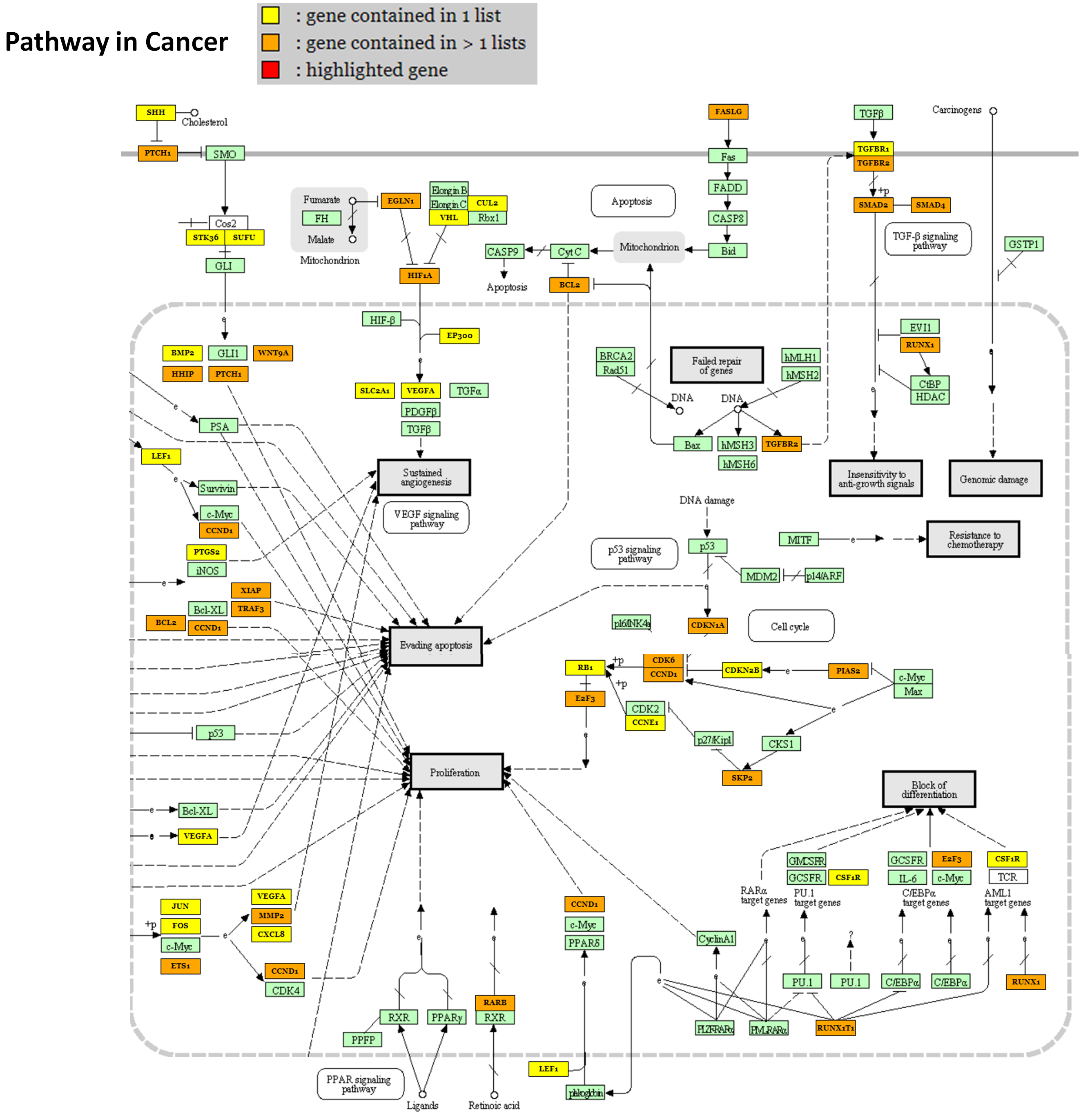

Supplement: Supplementary file 2 — Supplementary file2 (TIF 21291 KB) [file 12035_2021_2705_MOESM2_ESM.tif]
