# Supplementary material for: Cerebrospinal Fluid Extracellular Vesicles with Distinct Properties in Autoimmune Encephalitis and Herpes Simplex Encephalitis
Source: Mol Neurobiol. 2022 Jan 27;59(4):2441–55. doi: 10.1007/s12035-021-02705-2 (PMC9016041; doi:10.1007/s12035-021-02705-2)
Supplement: Supplementary file 3 — Supplementary file3 (DOCX 22 KB) [file 12035_2021_2705_MOESM3_ESM.docx]

Supplemental Table 2. Demographic features and symptoms of HSE negative.

| HSV negative Patient No. | 1 | 2 | 3 | 4 | 5 | 6 | 7 | 8 | 9 |
| --- | --- | --- | --- | --- | --- | --- | --- | --- | --- |
| Age (Year) | 25 | 19 | 31 | 33 | 39 | 34 | 39 | 34 | 30 |
| Sex | Male | Female | Male | Female | Male | Male | Female | Female | Male |
| Neurological syndrome |  |  |  |  |  |  |  |  |  |
| Decreased level of consciousness | - | - | - | - | + | - | - | - | - |
| Mental symptoms | - | + | - | - | - | - | - | - | - |
| fever | + | + | + | + | + | + | + | + | + |
| headache | + | + | + | + | + | + | + | + | + |
| seizure | - | - | - | - | + | - | - | - | - |
| focal sign | - | - | - | - | - | - | - | - | - |
| CSF analyses |  |  |  |  |  |  |  |  |  |
| Increased opening pressure | - | - | - | - | - | - | - | + | - |
| elevated white cells | - | - | - | - | - | - | - | - | - |
| elevated red cells | - | - | - | - | - | - | - | - | - |
| elevated protein | - | - | - | - | - | + | - | - | - |
| MRI T2 Lesions |  |  |  |  |  |  |  |  |  |
| temporal lobe | - | + | - | - | - | - | - | - | - |
| medial/inferior frontal lobe | - | - | - | - | - | - | - | - | - |
| cingulate/insula cortex | - | - | - | - | - | - | - | - | - |
| hippocampal | - | - | - | - | - | - | - | - | - |
| other | - | - | - | - | - | - | - | - | - |
| HSV PCR | - | - | - | - | - | - | - | - | - |

**Abbreviations**
CSF：cerebrospinal fluid
MRI：magnetic resonance imaging

HSV：herpes simplex virus
PCR：polymerase chain reaction
