# Supplementary material for: Cerebrospinal Fluid Extracellular Vesicles with Distinct Properties in Autoimmune Encephalitis and Herpes Simplex Encephalitis
Source: Mol Neurobiol. 2022 Jan 27;59(4):2441–55. doi: 10.1007/s12035-021-02705-2 (PMC9016041; doi:10.1007/s12035-021-02705-2)
Supplement: Supplementary file 4 — Supplementary file4 (DOCX 23 KB) [file 12035_2021_2705_MOESM4_ESM.docx]

**Supplementary table 1**. Biologic pathways enriched by differential CSF derived exosomal miRNAs in AE patients.

| KEGG pathway | p-value | #genes | #miRNAs |
| --- | --- | --- | --- |
| Fatty acid biosynthesis (hsa00061) | 3.8657E-14 | 6 | 4 |
| Mucin type O-Glycan biosynthesis (hsa00512) | 1.42349E-11 | 15 | 8 |
| Signaling pathways regulating pluripotency of stem cells (hsa04550) | 1.42349E-11 | 74 | 14 |
| Proteoglycans in cancer (hsa05205) | 4.50192E-11 | 91 | 15 |
| TGF-beta signaling pathway (hsa04350) | 6.51996E-10 | 46 | 11 |
| Pathways in cancer (hsa05200) | 2.31215E-07 | 160 | 15 |
| Hippo signaling pathway (hsa04390) | 4.86182E-06 | 68 | 13 |
| Glioma (hsa05214) | 1.29541E-05 | 32 | 13 |
| Wnt signaling pathway (hsa04310) | 1.56345E-05 | 63 | 14 |
| Ras signaling pathway (hsa04014) | 2.41546E-05 | 95 | 15 |
| Prostate cancer (hsa05215) | 2.58721E-05 | 46 | 14 |
| ErbB signaling pathway (hsa04012) | 9.92253E-05 | 42 | 13 |
| Pancreatic cancer (hsa05212) | 9.92253E-05 | 36 | 13 |
| mTOR signaling pathway (hsa04150) | 0.000179552 | 34 | 15 |
| Neurotrophin signaling pathway (hsa04722) | 0.000220794 | 55 | 14 |
| Axon guidance (hsa04360) | 0.00027181 | 56 | 13 |
| Melanoma (hsa05218) | 0.000407904 | 36 | 13 |
| Choline metabolism in cancer (hsa05231) | 0.000418229 | 49 | 13 |
| Endocytosis (hsa04144) | 0.000478265 | 86 | 14 |
| FoxO signaling pathway (hsa04068) | 0.000547234 | 60 | 14 |
| Rap1 signaling pathway (hsa04015) | 0.000554609 | 85 | 15 |
| Hepatitis B (hsa05161) | 0.000737595 | 55 | 15 |
| Colorectal cancer (hsa05210) | 0.001244673 | 31 | 11 |
| Ubiquitin mediated proteolysis (hsa04120) | 0.001364457 | 60 | 15 |
| Prolactin signaling pathway (hsa04917) | 0.001619692 | 34 | 12 |
| Dorso-ventral axis formation (hsa04320) | 0.001820194 | 17 | 11 |
| HIF-1 signaling pathway (hsa04066) | 0.002378915 | 47 | 15 |
| PI3K-Akt signaling pathway (hsa04151) | 0.002475088 | 125 | 15 |
| Regulation of actin cytoskeleton (hsa04810) | 0.002580914 | 82 | 13 |
| Renal cell carcinoma (hsa05211) | 0.00277084 | 32 | 13 |
| Adherens junction (hsa04520) | 0.002812758 | 37 | 13 |
| T cell receptor signaling pathway (hsa04660) | 0.003053472 | 47 | 12 |
| Acute myeloid leukemia (hsa05221) | 0.006711576 | 27 | 11 |
| Focal adhesion (hsa04510) | 0.009642196 | 79 | 14 |
| MAPK signaling pathway (hsa04010) | 0.009642196 | 95 | 15 |
| Basal cell carcinoma (hsa05217) | 0.012165771 | 26 | 8 |
| Insulin signaling pathway (hsa04910) | 0.01534755 | 56 | 14 |
| Protein processing in endoplasmic reticulum (hsa04141) | 0.016351868 | 67 | 13 |
| Hedgehog signaling pathway (hsa04340) | 0.017736945 | 24 | 8 |
| Transcriptional misregulation in cancer (hsa05202) | 0.018333206 | 63 | 12 |
| Small cell lung cancer (hsa05222) | 0.018333206 | 36 | 13 |
| Non-small cell lung cancer (hsa05223) | 0.018333206 | 25 | 13 |
| Long-term potentiation (hsa04720) | 0.018333206 | 30 | 14 |
| p53 signaling pathway (hsa04115) | 0.024353538 | 30 | 11 |
| B cell receptor signaling pathway (hsa04662) | 0.030493795 | 31 | 12 |
| Circadian rhythm (hsa04710) | 0.03092361 | 16 | 9 |
| Endometrial cancer (hsa05213) | 0.036253572 | 24 | 11 |
| Chagas disease (American trypanosomiasis) (hsa05142) | 0.049879165 | 40 | 15 |
